# Patterns of motor nerve demyelination and their prognostic significance in multifocal motor neuropathy

**DOI:** 10.1016/j.ibneur.2026.03.005

**Published:** 2026-03-13

**Authors:** Andreas Posa, Alexander Emmer, Tobias Biefel, Malte Erich Kornhuber

**Affiliations:** a University Clinic and Outpatient Clinic for Radiology, Martin Luther University Halle-Wittenberg, Halle, Germany; b Neurological Clinic, AKH Hospital Celle, Celle, Germany; c University Clinic and Outpatient Clinic for Neurology, Martin Luther University Halle-Wittenberg, Halle, Germany; d Neurological Clinic, HELIOS Hospital Sangerhausen, Sangerhausen, Germany

**Keywords:** Multifocal Motor Neuropathy (MMN), Intravenous Immunoglobulin (IVIg), Nerve Conduction Studies (NCS), Conduction Block (CB), Temporal Dispersion (TD)

## Abstract

**Background:**

Multifocal motor neuropathy (MMN) is a rare immune-mediated neuropathy characterized by motor nerve involvement and, typically, a good response to intravenous immunoglobulin (IVIg). However, a subgroup of patients shows poor or absent treatment response and a more rapid disease course. We aim to characterize clinical, laboratory, and electrophysiological features associated with IVIg response in MMN over a follow-up period of up to 20 years.

**Methods:**

Thirteen patients fulfilling diagnostic criteria for definite MMN were retrospectively analyzed. Detailed clinical data, comorbidities, IVIg treatment regimens, and serial nerve conduction studies (NCS) were evaluated. Particular attention was given to the extent and distribution of conduction block (CB) and temporal dispersion (TD). IVIg response was defined as ≥ 1-point improvement in MRC strength in at least two muscle groups within 4 weeks after infusion or equivalent functional improvement documented in medical records.

**Results:**

Four of 13 patients (31%) showed poor or absent IVIg response. These patients exhibited a higher number of clinically and electrophysiologically affected motor nerves at disease onset (median 6 vs. 3, p = 0.014) and more nerves with TD (median 2.5 vs. 1, p = 0.025), compared to IVIg responders. CB alone did not reliably distinguish responders from non-responders (median 1 vs. 1, p = 0.74). Anti-GM1 IgM antibodies were detected in 44% of the patients tested, of whom 75% were non-responders. Comorbidities (e.g., diabetes II, malignancy, autoimmune disease) were more frequent among non-responders.

**Conclusions:**

A pattern of early nerve involvement with prominent TD appears to be associated with a more aggressive disease course and poorer IVIg response. Diagnostic evaluation in MMN should not focus solely on focal CB but also systematically assess the number of nerves with TD. Prospective studies with standardized protocols are needed to validate these findings.

## Highlights

● Limited nerve involvement predicts favorable IVIg response.
● TD across multiple nerves at onset marks more severe disease.
● Adding TD assessment to CB improves prognostic stratification in MMN.

## 1 Introduction

Multifocal motor neuropathy (MMN) is a rare autoimmune polyneuropathy characterized by pure motor nerve fiber involvement, often affecting the upper limbs in an asymmetric, distal-predominant pattern (Claytor et al., 2025, Parry and Clarke, 1988, Pestronk et al., 1988). The disease typically shows a stepwise or slowly progressive course. Approximately 50% of patients have detectable IgM antibodies against GM1 ganglioside (Claytor et al., 2025, Pestronk et al., 1988, Cats et al., 2010a). Intravenous immunoglobulin (IVIg) is the established first-line therapy (Cats et al., 2010a, Li et al., 2024, Nobile-Orazio et al., 2002, Rajabally et al., 2025, Van den Berg-Vos et al., 2000). Although most patients respond favorably to IVIg, a clinically relevant subset does not (Nobile-Orazio et al., 2002, Van den Berg-Vos et al., 2000). Predictors of poor treatment response remain incompletely defined. Previous studies have suggested associations with longer disease duration, greater axonal loss, and less prominent or absent conduction block (CB) (Kuijf et al., 2005, Olney et al., 2003). In routine practice, diagnosis and disease monitoring rely heavily on motor nerve conduction studies (NCS). CB is a key electrophysiological hallmark of MMN. However, temporal dispersion (TD), reflecting more diffuse demyelination, may be underrecognized. Based on long-term follow-up of 13 MMN patients, we investigated whether patterns of TD and CB relate to disease course and IVIg response.

## 2 Methods

### 2.1 Patients and study design

Thirteen patients diagnosed with definite MMN according to established diagnostic criteria (Van den Berg-Vos et al., 2000, Olney et al., 2003) were retrospectively analyzed. All patients were evaluated and followed at the Department of Neurology, Martin Luther University Halle-Wittenberg, between 2003 and 2024. Follow-up duration ranged from 3 to 20 years (mean±SD: 12.5 ± 5.0 years). Clinical data were extracted from medical records, including age at onset, sex, clinical presentation, initial working diagnosis at referral (e.g. ALS, radiculopathy, entrapment syndromes), disease course, follow-up duration, and comorbidities. Patients were grouped according to the number of clinically affected motor nerves at disease onset (1–2 nerves vs. ≥ 3 nerves), reflecting the severity pattern used in subsequent analyses. Baseline and follow-up data were systematically compiled at the individual patient level and are summarized in Table 1.

**Table 1:** Summary of patient data.

**Patient**	**Age at Onset**	**Gender**	**Initial Diagnosis**	**Symptomatic Limb** **at Onset**	**Symmetry of Limb** **Involvement**	**Diffuse Hand Muscle** **Pareses at Onset**	**Mononeuritis (multi-** **plex) at Onset**	**Number of Diseased** **Nerves at Onset**	**Number of Nerves** **with CB at Onset**	**Number of Nerves** **with TD at Onset**	**5-Years Dynamics**	**CSF Total Protein** **at Onset (mg/l)**	**Presence of GM1-** **IgM at Onset**	**Response to IVIg**	**Other Diseases**
1	40	f	SE	Arm	unilateral	no	yes	1	1	0	constant	289	n.d.	yes	-
2	28	f	L5 R.itis	Leg	unilateral	no	yes	1	1	0	constant	n.d.	n.d.	yes	MS
3	61	f	MMN	Arm	unilateral	no	yes	2	2	1	constant	301	n.d.	yes	DM, MC
4	45	m	MMN	Arm	unilateral	no	yes	2	0	8	constant	572	negative	yes	-
5	52	m	MMN	Arm	unilateral	no	yes	3	1	0	constant	397	positive*	yes	DM
6	36	m	ALS	Arm & Leg	unilateral	no	yes	3	0	0	constant	438	negative	yes	-
7	43	m	C5/6 R.path.	Arm	bilateral	no	yes	4	0	2	constant	463	negative	yes	DM, PN
8	51	f	ALS	Arm & Leg	bilateral	no	yes	4	1	1	constant	211	negative	yes	-
9	48	f	MMN	Arm	bilateral	yes	no	5	1	1	progressive	446	positive*	no	SC
10	52	m	MMN	Arm & Leg	bilateral	no	yes	6	0	7	constant	843	negative	yes	-
11	29	m	Dist. SMA	Arm	bilateral	yes	no	6	0	2	progressive	938	positive*	no	-
12	37	m	MMN	Arm	bilateral	yes	no	6	1	7	progressive	816	n.d.	no	DM, AH
13	55	m	MMN	Arm & Leg	bilateral	yes	no	6	2	3	progressive	317	positive*	no**	DM, PC

Patients were grouped according to the number of diseased nerves at disease onset. CB, definitive conduction block. TD, temporal dispersion. MMN, multifocal motor neuropathy. ALS, amyotrophic lateral sclerosis. SE, supinator entrapment. L5 R.itis, L5 radiculitis. C5/6 R.path., C5/C6 radiculopathy. Dist. SMA, distale spinal muscle atrophy. MS, multiple sclerosis. DM, diabetes mellitus II. MC, mammary cancer. PN, plexus neuritis on magnetic resonance imaging. SC, squamous cell carcinoma of tongue. AH, autoimmune hepatitis. PC, prostate carcinoma. N.d., not done. *, anti-GM1-IgM normal. **, This patient showed considerable improvement to IVIg treatment three times after post surgery relapses.

### 2.2 Clinical data and IVIg treatment

IVIg therapy was administered at a standard dosage of 2 g/kg per treatment cycle over 1–2 days and repeated according to clinical response. Response to IVIg was defined as: a) improvement of ≥ 1 MRC grade in at least 2 clinically affected muscle groups within 4 weeks after treatment, or b) documented functional improvement in medical records (e.g., improvement in hand function, walking, or grip strength). The presence of anti-GM1 IgM antibodies was determined in 9 of 13 patients (n.d. = not done in 4 patients). Comorbidities, including type 2 diabetes mellitus, autoimmune diseases, and malignancies (e.g., breast cancer, prostate carcinoma, squamous cell carcinoma of the tongue), were systematically recorded due to their potential impact on disease course and nerve conduction findings. Magnetic resonance imaging (MRI) of the brachial or lumbosacral plexus was reviewed in selected patients to assess for plexopathies or structural lesions. These clinical and paraclinical variables are reported for each patient in Table 1.

### 2.3 Nerve conduction studies

Motor NCS were performed in a standardized manner. Uniform measurement techniques were applied, including predefined electrode placement, specified stimulation sites, and constant stimulation parameters. All examinations were conducted using equally calibrated equipment and under controlled conditions, including monitoring of skin temperature (at ≥ 32 °C). The procedures followed established neurophysiological guidelines to ensure high comparability and reproducibility of the results. The following motor nerves were examined depending on clinical involvement: ulnar, median, radial, peroneal, tibial, and musculocutaneous nerves. Stimulation was applied at distal, intermediate (below/above elbow, fibular head), and proximal sites (axilla, Erb’s point, popliteal fossa). Recording was performed from the corresponding target muscles using standard surface electrode placement. CB was defined as a > 50% CMAP amplitude or area reduction between proximal and distal stimulation sites without significant TD. TD was defined as a ≥ 30% increase in negative peak CMAP duration between distal and proximal stimulation, without meeting CB criteria. CB and TD were confirmed using supramaximal stimulation and by excluding technical factors such as inadequate stimulation, electrode misplacement, or temperature-related effects, in accordance with established neurophysiological guidelines. Diffuse TD was recorded when abnormal TD extended over ≥ 3 consecutive segments of the same nerve. Sensory NCS, F-wave studies, and inching techniques were performed when clinically indicated. Electrophysiological recordings and measurements were obtained and analyzed using standard clinical EMG/NCS systems with built-in analysis software and reviewed by experienced neurophysiological experts (A.E., M.E.K.). The number of tested nerves, the presence or absence of CB and TD, as well as anti-GM1 IgM antibody status, were documented for each patient at disease onset. These individual patient-level electrophysiological data are presented in Table 1.

### 2.4 Data analysis

For each patient, the number of clinically affected nerves, nerves with CB, and nerves with TD at onset and during follow-up were recorded. Descriptive statistics (median, range) were used due to small sample size. Exploratory comparisons between responders and non-responders were performed using Mann-Whitney U tests.

## 3 Results

### 3.1 Baseline characteristics

Thirteen patients were included in this study (5 female; mean age at onset 44.4 ± 10.0 years, range 28–61). The number of clinically affected nerves at onset ranged from 1 to 6 (median 4). At onset, weakness was unilateral in 6 and bilateral in 7 patients. Eight patients initially had upper limb involvement, 4 both upper and lower, and 1 lower limb only. Four patients presented with a diffuse, distal-predominant pattern at onset. Detailed patient-level data, including initial diagnosis, comorbidities, anti-GM1 IgM status, and electrophysiological findings, are summarized in Table 1.

### 3.2 Nerve conduction findings

**Fig. 1:**
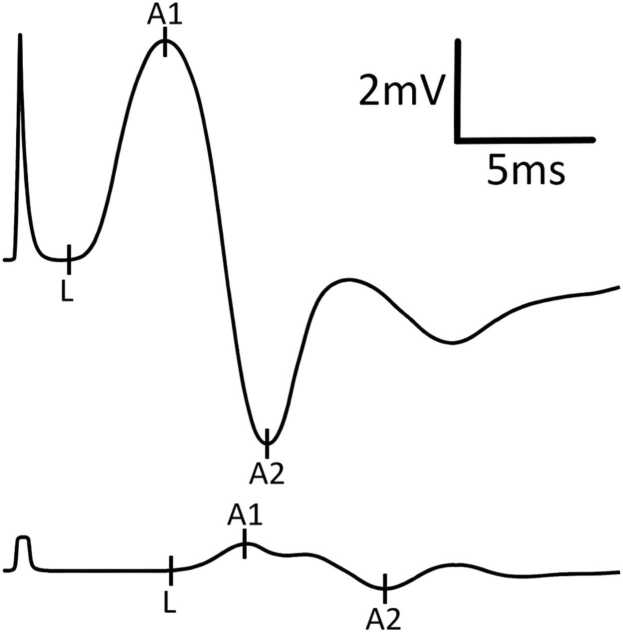
Definitive conduction block of a right radial nerve (patient #1). Onset latency (L), first negative peak (A1) and first positive peak (A2) are indicated. mV: millivolt; ms: milliseconds.

**Fig. 2:**
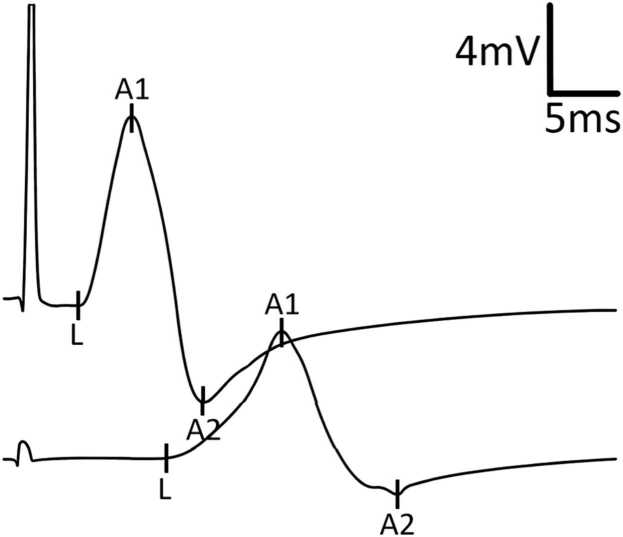
Temporal dispersion of a left median nerve (patient #9). Onset latency (L), first negative peak (A1) and first positive peak (A2) are indicated. mV: millivolt; ms: milliseconds.

**Fig. 3:**
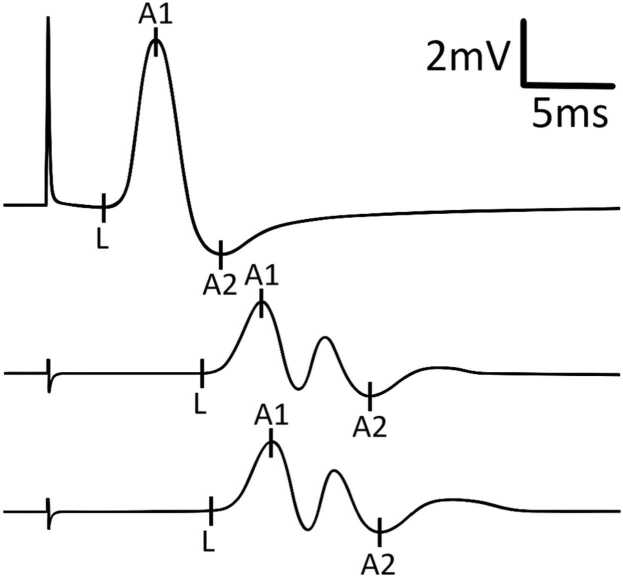
Example for presumably more focal temporal dispersion. Right peroneal nerve of an MMN patient (#10) stimulated above the ankle and below and above the fibular head. Onset latency (L), first negative peak (A1) and first positive peak (A2) are indicated. mV: millivolt; ms: milliseconds.

**Fig. 4:**
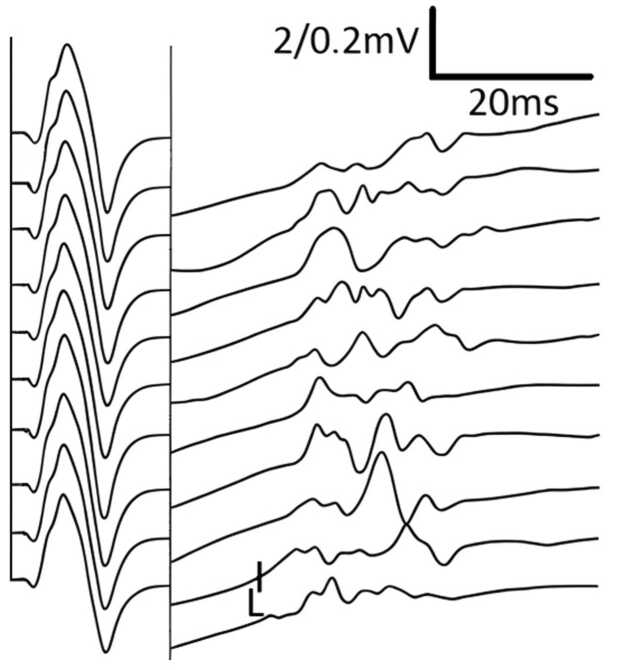
F-wave recordings of the right ulnar nerve of an MMN patient (#2; 167 cm in hight). Minimal latency (L) is marked in the 9th trace from top. Note the increase in temporal F-wave dispersion. mV: millivolt; ms: milliseconds.

**Fig. 5:**
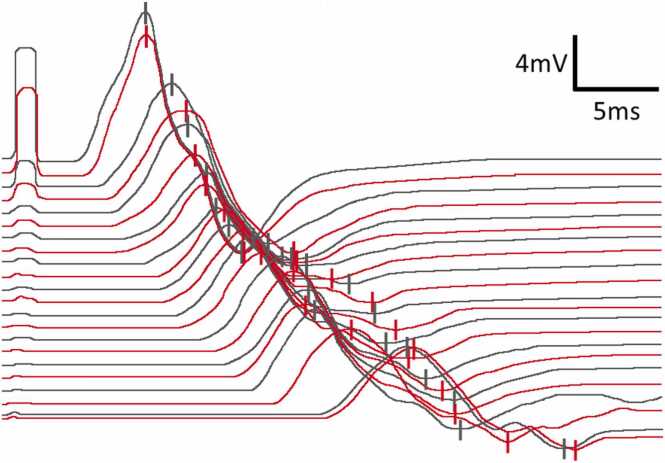
Inching of the right ulnar nerve of an MMN patient (#5) over almost the entire course. 2.5 cm nerve segments have been sampled between wrist (distalmost stimulus site) and axilla (second to proximal most stimulus site). The most proximal stimulus was set over Erb‘s point. Note the gradual amplitude decline with usually minor changes between any 2 neighbouring sites. Although patient #5 responded well to IVIg, this figure is shown to illustrate the electrophysiological appearance of diffuse temporal dispersion. Traces were color-coded for improved visual clarity; raw data were not altered. The first negative and the first positive peak are each marked. mV: millivolt; ms: milliseconds.

Initial NCS demonstrated definitive CB in: a) 2 nerves in 2 patients, b) 1 nerve in 6 patients, and c) none in 5 patients (illustrative example in Fig. 1). TD (without CB) was present in 9 patients, affecting 1–8 nerves per patient (illustrative example in Fig. 2). TD was often diffuse rather than focal (illustrative examples in Fig. 3, Fig. 4, Fig. 5). During follow-up, all patients developed at least one nerve with partial CB.

### 3.3 IVIg response and electrophysiological patterns

Nine patients (69%) were classified as IVIg responders. Four patients (31%) showed poor or absent response despite standard dosing. Non-responders had a higher median number of clinically involved nerves at onset (median 6 vs. 3, p = 0.014) and more nerves with TD (median 2.5 vs. 1, p = 0.025). CB distribution was similar in both groups (median 1 vs. 1, p = 0.74). One non-responder exhibited a reproducible response to IVIg after disease relapse triggered by surgery. Comorbidities were present in 5 patients (diabetes mellitus II), 3 had malignancies, and 2 other autoimmune diseases. IVIg response was observed in 5 of 6 patients without comorbidities (83%) and in 4 of 7 patients with comorbidities (57%). Response rates in subgroups were 60% (3/5) in patients with diabetes mellitus II, 33% (1/3) in patients with malignancies, and 50% (1/2) in patients with other autoimmune diseases.

### 3.4 Laboratory findings

Of the 13 patients, 9 were tested for anti-GM1-IgM antibodies, of whom 4 were positive (44%). Three of the anti-GM1-IgM-positive patients were IVIg non-responders (75%), whereas 1 showed a favorable response to IVIg therapy. The total mean CSF protein level was 503 ± 240 mg/L (range: 211–938 mg/L). For IVIg responders, the mean value was 439 mg/l (range: 211–843 mg/l), for IVIg non-responders the mean value was 629 mg/l (range: 317–938 mg/l).

## 4 Discussion

### 4.1 Clinical relevance of nerve involvement patterns

This study provides novel insights into the prognostic and therapeutic relevance of nerve involvement patterns in MMN. While CB has traditionally been considered the hallmark electrophysiological feature supporting MMN diagnosis (Claytor et al., 2025), our findings emphasize the critical role of TD and its distribution along motor nerves in determining both disease course and response to IVIg therapy. In our cohort of 13 patients followed over periods ranging from 3 to 20 years, a subset of 4 patients (31%) showed poor response to IVIg. According to current studies, the non-responder rate is approximately 6–30% (Claytor et al., 2025). In our cohort, these patients displayed more motor nerve involvement at disease onset and more nerves with TD.

### 4.2 Limitations of conduction block as a prognostic marker

Our data suggest that CB alone is insufficient to stratify patients according to prognosis or likelihood of IVIg response. While CB was observed in most patients during follow-up, its presence did not differentiate responders from non-responders. This finding is consistent with previous reports indicating that the clinical response to IVIg may be less pronounced in patients with only possible or probable CB (Nobile-Orazio et al., 2002, Léger, 2014, Van der Pol et al., 2010). In our cohort, the early presence of TD at disease onset, rather than the presence of CB, appeared to be more closely associated with disease severity and functional deterioration, underscoring the limited predictive value of CB as a standalone marker.

### 4.3 Temporal dispersion as an indicator of widespread demyelination

In our cohort, TD emerged as a more informative electrophysiological feature for disease progression and IVIg response, manifesting either as CMAP widening or as pronounced dispersion with amplitude reduction. These observations support the interpretation by Van den Berg-Vos et al., who suggested that some CBs may result from phase cancellation due to TD rather than true conduction block (Van den Berg-Vos et al., 2000). Diffuse TD likely reflects widespread demyelination along the nerve, which can contribute to CMAP amplitude loss traditionally attributed to axonal degeneration. Therefore, TD may represent an intermediate electrophysiological marker between pure demyelination and secondary axonal damage, providing a mechanistic explanation for the progressive functional decline seen in certain patients.

### 4.4 Correlation of TD with IVIg response

The early presence of TD, regardless of its quantitative extent and independent of CB, was observed in all patients in our cohort who subsequently exhibited a progressive disease course and poor IVIg response. In contrast, TD was absent at disease onset in almost half of the patients with stable disease and good IVIg responsiveness. Importantly, our data do not support a linear relationship between the extent of TD and treatment response. Rather, TD at disease onset appears to represent a qualitative marker of an unfavorable disease phenotype than a dose-dependent predictor of IVIg efficacy.

### 4.5 Implications for diagnosis and disease monitoring

The distinction between CB and TD has important diagnostic and prognostic implications. Reliance solely on CB may underestimate the extent of nerve involvement and delay accurate diagnosis, as exemplified by two of our patients (15%) initially misdiagnosed with motor neuron disease, a scenario reported in up to 32% of MMN cases (Cats et al., 2010a, Kleinveld et al., 2024). In our cohort, only 7 of 13 patients (54%) initially received the correct diagnosis of MMN. The rarity of MMN and its clinical overlap with other disorders are associated with high initial misdiagnosis rates, which have been reported to reach up to 87% in some studies, leading to substantial delays in correct diagnosis of up to 6 years (Claytor et al., 2025). Incorporating TD assessment into routine nerve conduction studies may provide earlier recognition of widespread demyelination, inform prognosis, and guide treatment strategies. The presence of TD at disease onset may provide clinically relevant contextual information beyond CB alone, supporting earlier risk stratification and more individualized management strategies in routine practice.

### 4.6 Potential additional predictors of IVIg response

The focus of this study was on electrophysiological patterns of motor nerve demyelination and their prognostic significance in MMN. In addition, 44% of tested patients were positive for anti-GM1 IgM antibodies. Consistent with previous reports, approximately 50% of MMN patients have detectable IgM antibodies against GM1 ganglioside (Claytor et al., 2025, Pestronk et al., 1988, Cats et al., 2010a), which show relatively high specificity for MMN (93%) (Nobile-Orazio et al., 2014), although their absence does not exclude the diagnosis. Patients with anti-GM1 IgM antibodies have been reported to exhibit more severe weakness, greater disability, and increased axonal loss compared to seronegative patients (Cats et al., 2010b). In our cohort, 75% of anti-GM1 IgM-positive patients were IVIg non-responders. However, to date, there is no evidence that seropositive patients respond worse to IVIg than seronegative patients, and the limited sample size of our study precludes any definitive conclusion.

In our MMN cohort, the mean CSF total protein level was 503 ± 240 mg/L (range 211–938 mg/L). This is comparable to the population-based mean CSF protein level (522 ± 184 mg/L) and largely within the 95% reference interval (240–934 mg/L) reported in a large community-based study (Fautsch et al., 2023), which is broader than clinically applied upper reference limits (95th percentile: 530–690 mg/L) (Hegen et al., 2016). Current literature is consistent with our findings, indicating that CSF protein levels in MMN are usually normal or, at most, mildly elevated (Claytor et al., 2025). In our cohort, IVIg non-responders exhibited higher mean CSF protein levels compared with IVIg responders (629 vs. 439 mg/L). However, substantial overlap between groups and the small sample size preclude definitive conclusions, rendering this observation hypothesis-generating rather than confirmatory. To our knowledge, there are currently no published studies demonstrating that IVIg non-responders have significantly higher CSF protein levels compared to responders. Existing literature specifically notes that elevated CSF protein does not correlate with response to IVIG in MMN (Claytor et al., 2025).

In our MMN cohort, comorbidities included diabetes mellitus II, malignancies (mammary cancer, squamous cell carcinoma of the tongue, prostate carcinoma), and other autoimmune diseases (multiple sclerosis, autoimmune hepatitis). The co-occurrence of MMN with a diabetic metabolic state has been reported only in very rare case reports (Reisin et al., 2005), and evidence for an epidemiological association is lacking. Similarly, there are only a few case reports regarding malignancies in MMN (Garcia-Moreno et al., 2004, Stern et al., 2006, Adebayo et al., 2025), which does not allow for a consistent epidemiological classification. The occurrence of MMN as a paraneoplastic syndrome appears to be extremely rare (Zoccarato et al., 2021), and data on the specific malignancies observed in our cohort are currently unavailable. Regarding other autoimmune diseases, MMN patients show a higher prevalence, particularly for type 1 diabetes, Hashimoto's thyroiditis, ankylosing spondylitis, Crohn's disease, and celiac disease, suggesting a possible shared autoimmune diathesis (Claytor et al., 2025, Cats et al., 2012). For the autoimmune diseases multiple sclerosis and autoimmune hepatitis observed in our cohort, no direct pathophysiological relationships have been found in the literature, although a familial autoimmune predisposition has been discussed (Cats et al., 2012). With regard to IVIg response, our data suggest that patients without comorbidities tended to respond better than those with comorbidities (83 vs. 57%). However, due to the small sample size, these observations are purely descriptive and not statistically conclusive.

### 4.7 Limitations

This retrospective study is limited by a small cohort size and non-standardized timing of electrophysiological assessments, as measurements were performed according to routine clinical care rather than a predefined follow-up schedule. Although the electrophysiological measurements themselves were conducted using standardized techniques, some data (e.g., MRC scores, antibody testing) were incomplete. Statistical analyses were exploratory only. These factors limit the generalizability of our findings to the broader MMN population. Additionally, the variability in follow-up duration and clinical documentation may have influenced the assessment of IVIg response and electrophysiological patterns. Prospective multicenter studies with standardized protocols, larger patient cohorts, and systematic TD assessment are needed to confirm the prognostic role of temporal dispersion in MMN and to better define its utility in guiding individualized treatment strategies.

## 5 Conclusions

In this cohort of 13 MMN patients, a pattern of temporally dispersed motor nerve involvement at disease onset was observed in both IVIg responders and non-responders. Non-responders, however, exhibited more frequent TD, which was associated with a more aggressive clinical course and a poorer response to IVIg therapy. In contrast, patients with limited nerve involvement generally responded well to IVIg. Our findings suggest that CB alone may not fully capture disease severity or predict therapeutic response. We therefore propose that systematic evaluation of TD should complement CB analysis in routine MMN diagnostics. Early identification of patients with extensive TD could facilitate risk stratification, enable closer clinical monitoring, and support individualized treatment strategies. These results underscore the potential of TD as a prognostic electrophysiological marker and highlight the need for prospective studies to validate its utility in guiding therapeutic decision-making.

## Ethics statement

Ethics Committee of the Medical Faculty of the Martin Luther University Halle-Wittenberg. Positive decision from February 7, 2024. Processing number: 2024–027.

## Funding

The publication of this article received financial support from the Open Access Publication Fund of the Martin Luther University Halle-Wittenberg.

## CRediT authorship contribution statement

**Tobias Biefel:** Writing – review & editing, Methodology, Investigation. **Alexander Emmer:** Writing – review & editing, Methodology, Investigation, Formal analysis, Data curation. **Malte Erich Kornhuber:** Writing – review & editing, Supervision, Resources, Methodology, Investigation. **Andreas Posa:** Writing – original draft, Visualization, Validation, Methodology, Investigation, Formal analysis, Data curation, Conceptualization.

## Declaration of Competing Interest

The authors (Andreas Posa, Alexander Emmer, Tobias Biefel, Malte Erich Kornhuber) declare that they have no known competing financial interests or personal relationships that could have appeared to influence the work reported in this paper.
